# Relationship Between Poor Sleep and Depressive Symptoms in Korean Elite Youth Athletes

**DOI:** 10.3390/jcm14020479

**Published:** 2025-01-14

**Authors:** Youngju Choi, Seoyeon Kim, Soo-Hyun Park, Kitae Kim, Rye Kyeong Kim, Hyo-Bum Kwak, Jinkyung Cho

**Affiliations:** 1 Institute for Specialized Teaching and Research (INSTAR), Inha University, Incheon 22212, Republic of Korea; choiyoungju0323@gmail.com; 2 Department of Kinesiology, Inha University, Incheon 22212, Republic of Koreakwakhb@inha.ac.kr (H.-B.K.); 3 Institute of Sports & Arts Convergence (ISAC), Inha University, Incheon 22212, Republic of Korea; 4 Department of Sport Science, Korea Institute of Sport Science, Seoul 05540, Republic of Korea; otajulia@kspo.or.kr (S.-H.P.); 71eh@kspo.or.kr (K.K.);; 5 Department of Human Movement Science, Incheon National University, Incheon 22012, Republic of Korea; 6 Department of Biomedical Science and Engineering, Inha University, Incheon 22212, Republic of Korea; 7 Department of Sport Science, Sungkyunkwan University, Suwon 16419, Republic of Korea

**Keywords:** adolescents, elite sports, mental health, Pittsburgh Sleep Quality Index

## Abstract

**Background**: Sleep and mental health are crucial to elite youth athletes, who face combined pressures of training, competition, school, and social commitments. We examined the association between sleep and depression in elite youth athletes. **Methods**: We analyzed data of 248 Korean world-class youth athletes (aged 13–19 years). The athletes completed the Pittsburgh Sleep Quality Index (PSQI), the Morningness–Eveningness Questionnaire (MEQ), and the Center for Epidemiologic Studies Depression Scale (CES-D). **Results**: Overall, 50.4% of the athletes were considered poor sleepers (global PSQI score ≥ 5.5); 23.8% reported depressive symptoms (CES-D score ≥ 16). Depression was 4.26 times (95% confidence interval [CI], 2.00–9.09, *p* < 0.001) more likely in poor than in good sleepers. Odds of depression were increased with poor subjective sleep quality (odds ratio [OR]: 4.62; 95%CI, 2.10–10.18, *p* < 0.001), prolonged sleep latency (OR: 2.45: 95% CI, 1.28–4.69, *p* < 0.01), increased sleep disturbances (OR: 3.98: 95% CI, 1.83–8.63, *p* < 0.001), and daytime dysfunction (OR: 3.28; 95% CI, 1.67–6.44, *p* < 0.001). Depressive symptoms were associated with worse sleep, particularly poor subjective sleep quality, prolonged sleep latency, increased sleep disturbances, and increased daytime dysfunction. **Conclusions**: These results suggest that depressive symptoms are associated with poor sleep in the elite youth athlete population.

## 1. Introduction

During adolescence, which is a critical stage of development characterized by significant physiological and psychosocial changes, sleep is crucial for overall physical and psychological health, including physical development and brain function [1]. This is particularly true for youth athletes, as sleep is vital not only for recovery and adaptation but also for meeting the demands of intensive training and competition. Additionally, these athletes acquire a wide range of motor skills related to actual performance, from fundamental movements to highly specialized and complex motor skills, highlighting the importance of sleep in consolidating motor skill learning. Given these physical needs, youth athletes may require more than 10 h of sleep per night, exceeding the general recommendation for teenagers (8–10 h) [2,3]. 

Adult athletes across various sports often have suboptimal sleep profiles [4], but few studies have examined the sleep patterns of youth athletes [5]. A recent study of Singapore’s elite national youth athletes reported an average of 7 h of sleep per night, with 45.2% experiencing poor sleep quality [6], which is below the estimated basal sleep requirement of 8–10 h for adolescents [2,3]. 

Similar to those in the general population, sleep problems in athletes are also related to mental health [7], which has a unique influence on training, recovery, and athletic performance [4]. The relationship between sleep and mental health may be bidirectional [8], suggesting that insufficient sleep contributes to the developmental process of a reciprocal negative cycle between sleep and mental health. Depression, in particular, has been noted to alter all sleep parameters [9]. Sleep also represents a core component of depression in adolescence [10]. 

Elite youth athletes, who face combined pressures of training, competition, school, and social commitments, may be particularly vulnerable to insufficient sleep and depression, both of which influence athletic training and performance. However, few studies have explored the relationship between sleep and mental health, and they focused on elite adult athletes [7]. Understanding these relationships can guide the development of education and implementation of sleep hygiene and mental well-being programs in this population [11,12]. 

Therefore, this study aimed to examine the association between sleep and depression in elite adolescent athletes using data from the 2023 Next Generation Project of South Korea, which was a cross-sectional study conducted among Korean world-class youth athletes. We hypothesized that elite youth athletes reporting higher levels of global sleep dysfunction (i.e., poor sleepers) would also report more depressive symptoms than good sleepers.

## 2. Materials and Methods

### 2.1. Participants 

**Figure 1:**
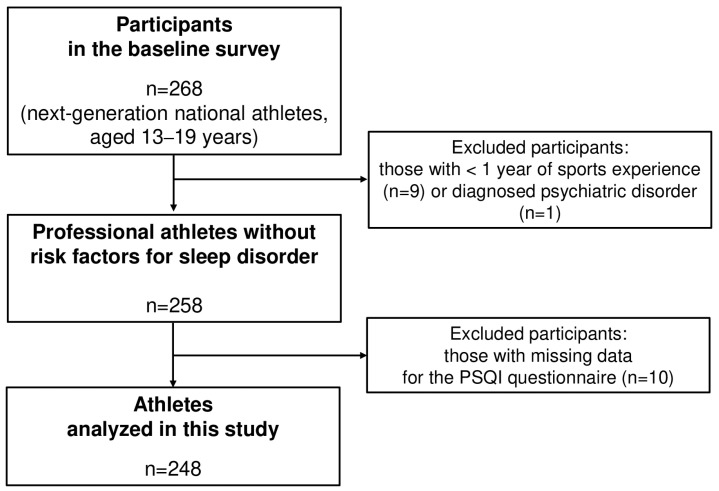
Flowchart for inclusion and exclusion of study participants.

A total of 268 athletes (age: 16.4 ± 0.1 years; range: 13–19 years) who participated in the Korea Junior National Team training camp were recruited for this study. The athletes participated in the following sports: handball (*n* = 32), badminton (*n* = 60), weightlifting (*n* = 19), short-track skating (*n* = 45), speed skating (*n* = 30), figure skating (*n* = 22), wrestling (*n* = 25), and cycling (*n* = 35). Athletes with a diagnosed psychiatric disorder (e.g., autism) (*n* = 1) or less than 1 year of sports experience (*n* = 9) were excluded. Of the 258 athletes, 10 athletes were excluded due to missing data on the Pittsburgh Sleep Quality Index (PSQI). Thus, a total of 248 participants were included in the final data analysis (Figure 1). 

All athletes and their parents provided written informed consent prior to participation. The study was conducted in accordance with the Declaration of Helsinki and was approved by the Institutional Review Board of Korea Institute of Sports Science (KISS-23020-2307-02).

### 2.2. Sample Size Estimation

The sample size was determined based on a previous study [13] indicating that poor subjective sleep quality is associated with depressive symptoms in adolescents. Considering an effect size of 0.41 (*r*), a detection power of 0.95, and an α level of 0.05, the required sample size was calculated as 71 participants, based on the use of a bivariate normal model in G*Power 3.1 (Heinrich Heine, Dusseldorf, Germany). Overall, 268 athletes were recruited for this study. 

### 2.3. Procedures

This study used a cross-sectional design with a large sample of elite youth athletes from the 2023 Korea Next Generation Project. The participants completed questionnaires covering demographic and athletic information (including items on age, sports career, and medical history), as well as health questionnaires (including items on sleep habits, quality, chronotype, and depression), in a quiet location, in the presence of the investigator. The participants then underwent anthropometric measurements. Data collection was conducted on different days to accommodate the training schedule of each sport.

### 2.4. Sleep Variables

Global sleep dysfunction was evaluated using the validated Korean version of the self-administered PSQI questionnaire (PSQI-K) [14]. The PSQI assesses sleep quality over the past month [15] and consists of seven components: subjective sleep quality (satisfaction with daily sleep), sleep latency (time taken to transition from full wakefulness to sleep), sleep duration, habitual sleep efficiency (proportion of hours asleep to total hours in bed), sleep disturbances, sleep medication consumption habits, and daytime dysfunction (difficulty staying awake during activities). Each component was scored on a scale of 0–3, with higher scores representing worse sleep quality. Subjective sleep quality was rated from very good (0) to very poor (3). Sleep latency, based on the time taken to fall asleep, ranged from <15 min (0) to >60 min (3). Sleep duration was rated as >7 h (0), 6–7 h (1), 5– 6 h (2), or <5 h (3). Habitual sleep efficiency, the percentage of time spent sleeping in bed, was scored from ≥85% (0) to <65% (3). Sleep disturbance was evaluated by frequency, ranging from rare (0) to frequent (3) occurrences. The use of sleep medication ranged from none (0) to three or more times per week (3). Daytime dysfunction, including difficulty staying awake or maintaining enthusiasm during daily activities, was rated on a scale ranging from no trouble (0) to frequent issues (3).

The global PSQI score was calculated as the sum of the seven component scores, with the total score ranging from 0 to 21. Higher scores indicated worse sleep quality. A cutoff score of 5.5 was used to classify participants as either “good sleepers (<5.5)” or “poor sleepers (≥5.5)” [15].

### 2.5. Chronotype Questionnaire

The Morningness–Eveningness Questionnaire (MEQ) was used to characterize the circadian typology of each participant [16]. The Korean version of the MEQ has good internal consistency and validity [17]. The MEQ comprises 19 questions, with higher scores indicating higher morningness and lower eveningness. Based on their scores, each participant was classified as a morning type (score: 59–86), intermediate type (score: 42–58), or evening type (score: 16–41).

### 2.6. Assessments of Depressive Symptoms

Depressive symptoms were evaluated using a 20-item version of the Center for Epidemiological Studies Depression Scale (CES-D), which is a self-administered questionnaire assessing depressive symptoms experienced during the most recent 1 week, including depressed affect (e.g., “I felt depressed”), lack of positive affect (e.g., “I enjoyed life”; reverse-coded), somatic symptoms (e.g., “I did not feel like eating”), and interpersonal difficulties (e.g., “I felt that people dislike me”). Higher scores indicate a more depressed mood. Participants were asked about their mental state in the previous week using a four-point Likert scale ranging from 0 to 3: rarely or never (<1 day), sometimes (1–2 days), often (3–4 days), or most of the time (5–7 days). The CES-D is a valid tool for measuring the mental health of individuals, describing a wide range of depressive symptoms. It has also been validated as a reliable and effective tool for assessing depression symptoms in Korean adolescents [18]. The total score ranges from 0 to 60, with higher scores indicating greater levels of depressive symptoms. Among adolescents, a CES-D score of 16 or higher is considered indicative of a possible depressive disorder [19]; thus, a cutoff CES-D value of ≥16 points was used to classify participants as having “depressive symptoms” in this study.

### 2.7. Statistical Analysis

The Shapiro–Wilk test was conducted to evaluate the normality of all parameters. Continuous variables, whether normally distributed or not, were presented as the mean ± standard error. Categorical variables were presented as the numbers in the group and percentages of the total. Statistical significance between good and poor sleepers was evaluated using the Pearson χ^2^ test for categorical variables and either the independent *t*-test (for parametric) or the Mann–Whitney U test (for non-parametric) for continuous variables, as appropriate. To assess the association between sleep quality (measured by the PSQI) and depression, we performed an analysis of covariance (ANCOVA) and logistic regression. ANCOVA was used to compare CES-D scores between good and poor sleepers, adjusted for age, sex, body mass index (BMI), sports, and length of athletic career. Logistic regression analysis was performed to estimate odds ratios (ORs) and the corresponding 95% confidence intervals (95%CIs) for the relationship between depression and poor sleep using the global PSQI score and its seven components, adjusted for plausible confounding factors, such as age, sex, sport, and length of athletic career. For logistic regression analyses of PSQI components, subscale scores of 0 and 1 were coded as 0, and scores of 2 and 3 were coded as 1. 

All analyses were performed using IBM SPSS Statistics 29 (SPSS Inc., Chicago, IL, USA), with statistical significance set at *p* < 0.05. 

## 3. Results

### 3.1. Characteristics of All Participants

The characteristics of all participants stratified by sleep quality are shown in Table 1. Sleep quality was categorized into two groups based on the global PSQI score: <5.5 (good sleepers) and ≥5.5 (poor sleepers). Among the 248 study participants (48% female, mean age 16.4 ± 0.1 years), 128 (50.4%) were classified as poor sleepers, and 59 individuals reported depressive symptoms. 

**Figure 2:**
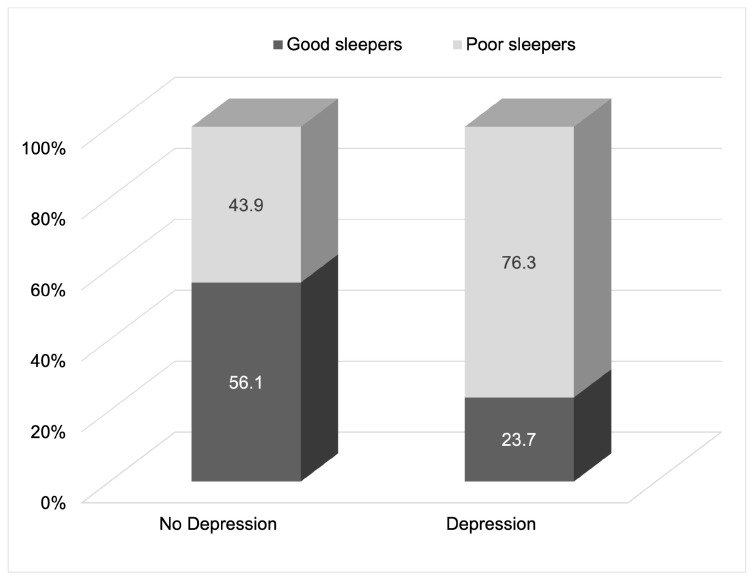
The prevalence of poor sleepers (global PSQI score ≥ 5.5) in groups with and without depressive symptoms. The χ^2^ statistical test was performed to assess differences in the prevalence of poor sleepers (defined as a global PSQI score of ≥5.5) between the groups with and without depressive symptoms (*p* < 0.001).

There were significant differences in terms of age, length of athletic career, and CES-D scores between good and poor sleepers. The prevalence of depressive symptoms among poor sleepers was high (35.2%), significantly exceeding that among good sleepers (11.7%, *p* < 0.001). Individuals with depressive symptoms had a higher prevalence of poor sleep than those without depressive symptoms (76.3% vs. 43.9%, *p* < 0.001; Figure 2). 

### 3.2. Comparison of Sleep Parameters According to the Presence of Depressive Symptoms

Significant differences in sleep parameters, as assessed by the global PSQI score and its components, between the non-depressed and depressed groups are shown in Table 2. The mean global PSQI score (5.24 ± 0.19 without depressive symptoms vs. 7.22 ± 0.36 with depressive symptoms; *p* < 0.001) and PSQI sub-component scores of subjective sleep quality (0.93 ± 0.04 vs. 1.34 ± 0.08, *p* < 0.001), sleep latency (0.98 ± 0.06 vs. 1.47 ± 0.14, *p* < 0.005), sleep disturbance (0.98 ± 0.04 vs. 1.32 ± 0.09, *p* < 0.001), and daytime dysfunction (1.23 ± 0.06 vs. 1.80 ± 0.10, *p* < 0.001) were significantly higher in participants with depressive symptoms than in those without depressive symptoms. 

### 3.3. Odds Ratio of Depressive Symptoms by PSQI and Its Components

**Figure 3:**
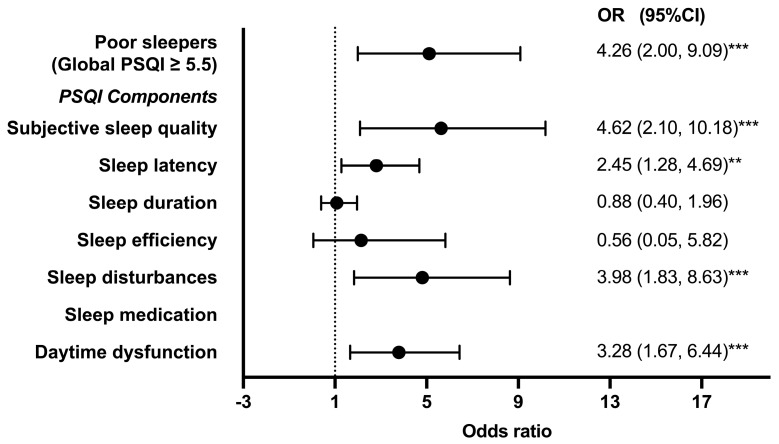
Odds ratio values for depressive symptoms by PSQI and its components. Data are presented as the adjusted odds ratio (95% CI) for the prevalence of depressive symptoms. CI, confidence interval; OR, odds ratio; PSQI; Pittsburgh Sleep Quality Index. The logistic regression analysis was conducted after adjusting for age, sex, BMI, sport, and length of athletic career. ** *p* < 0.01, *** *p* < 0.001.

The results of the logistic regression analysis examining the associations between sleep (global PSQI and its components) and depressive symptoms after adjusting for age, sex, sport, and length of athletic career are shown in Figure 3. The odds ratio of depression among poor sleepers with a global PSQI score of ≥5.5 was 4.26 (95%CI, 2.00–9.09, *p* < 0.001) compared to good sleepers, whose PSQI scores were <5.5. Specifically, the odds ratio of depression among the athletes with poor subjective sleep quality, sleep latency, sleep disturbances, and daytime dysfunction were 4.62 (95%CI, 2.10–10.18, *p* < 0.001), 2.45 (95%CI, 1.28–4.69, *p* < 0.01), 3.98 (95%CI, 1.83–8.63, *p* < 0.001), and 3.28 (95%CI, 1.67–6.44, *p* < 0.001), respectively. 

## 4. Discussion

This study aimed to determine the association between sleep quality and depressive symptoms in elite youth athletes. This study identified a strong association between poor sleep, defined as a global PSQI score of ≥5.5, and an increased risk of depressive symptoms. Additionally, we observed significant associations between depressive symptoms and several sleep parameters, including poor subjective sleep quality, prolonged sleep latency, sleep disturbances, and daytime dysfunction. These findings are crucial for elite youth athletes, as both sleep quality and mental health can affect performance, injury risk, overall well-being, and academic success.

With growing concerns about sleep and mental health in adolescents, research has increasingly focused on the relationship between sleep duration and depressive symptoms, despite inconsistent findings [20,21,22,23]. Several meta-analyses have shown that the association between sleep and depression varies considerably, depending on the different sleep measurements used. These findings suggest that sleep duration alone may not fully explain the relationship between sleep and depressive symptoms in adolescents. In contrast, assessing sleep perception, such as global sleep quality, provides a more reliable and informative indicator of the association between sleep and depressive symptoms than measuring sleep behavior (i.e., sleep quantity) [13]. Notably, a recent study by Suppiah et al. highlighted that sleep quality, as measured using the PSQI, is strongly influenced by factors such as specific sports participation, training, and sleep hygiene habits in elite youth athletes [24]. Additionally, our recent study showed that poor sleepers, as identified by the PSQI, exhibited decreased sleep quality (i.e., increased sleep latency, waking after sleep onset, and decreased sleep efficiency) as measured by actigraphy [25]. These results support the possibility that the PSQI accurately reflects objective actigraphy-measured sleep parameters. Based on these findings, the PSQI was used to determine sleep quality in this study. In addition to the high prevalence of poor sleep in elite youth athletes, our study revealed that depressive symptoms were associated with worse sleep, specifically regarding poor subjective sleep quality, increased sleep latency, increased sleep disturbances, and increased daytime dysfunction. Therefore, the global PSQI score, a widely recognized indicator of overall sleep quality, may serve as a superior tool for identifying the risk of depressive symptoms in youth athletes. 

Although many youth athletes and coaches recognize that adequate sleep is essential for recovery and optimal athletic performance, various factors, such as intensive training demands, competition, and societal pressures (e.g., early school and sports practice schedules), often interfere with sleep. Evidence suggests that the incidence of mental health issues, such as depression, may also increase due to increased training demands, competition, and both internal and external pressures from the elite sports environment [26]. Compared to their nonathletic peers, elite youth athletes face additional psychological stress from sports directors, coaches, parents, competitors, and self-imposed expectations [27]. Indeed, our study revealed that the prevalences of poor sleep and depressive symptoms were 50.4% and 23.8%, respectively, which was consistent with the findings of previous studies conducted among elite youth athletes [6,28]. During adolescence, changes in bioregulatory sleep processes can lead to various sleep difficulties [29], such as difficulty falling asleep, sleep disturbances, and daytime dysfunction, which, in turn, contribute to increased depression. A meta-analysis has suggested that prolonged sleep latency and sleep disturbances, which are related to wakefulness in bed, are characteristics of depression in adolescents [30]. Our findings further support this beyond the general population, showing that elite youth athletes with depressive symptoms exhibited significantly prolonged sleep latency, poor sleep quality, increased sleep disturbances, and increased daytime dysfunction, even after adjustments for potential confounders, such as age, BMI, sport, and length of athletic career. Taken together, these findings underscore the importance of understanding specific sleep problems when determining the relationship between sleep and mental health, particularly in elite youth athletes. 

The mechanisms underlying the relationship between sleep quality and depressive symptoms in youth athletes remain to be elucidated. The relationship between sleep quality and depressive symptoms may be bidirectional, particularly during adolescence: poor sleep acts as a risk factor for depression, which, in turn, exacerbates poor sleep [31]. Poor sleep and depression in adolescents have been suggested to be associated with reduced prefrontal cortex activity and increased limbic system activity [32], both of which are involved in negative emotions [33]. Emotional regulation, a key risk factor in mitigating negative psychological outcomes [34], is impaired by poor sleep quality, as it reduces activity in brain regions that are essential for processing emotions upon waking. This, in turn, can lead to hyperarousal, which delays the onset of sleep. Indeed, our study demonstrated that athletes with depressive symptoms exhibited significantly higher levels of daytime dysfunction, along with sleep problems such as prolonged sleep latency, poor sleep quality, and increased sleep disturbances. In addition, delayed circadian timing, which can affect multiple sleep dimensions, such as chronotype, sleep debt, and social jet lag, may also contribute to adolescent depression [35]. However, our results showed that most athletes had an intermediate chronotype, and no significant associations were seen between chronotype and depression, although the general adolescent population with elevated depressive symptoms often reports having a later chronotype [36] and a preference for evening activities [37,38]. Further research is necessary to elucidate the mechanisms linking sleep quality and depressive symptoms in elite youth athletes. 

Our study has some limitations. First, we could not determine a causal relationship between sleep and depression because of the cross-sectional study design. Future longitudinal research is needed to confirm the associations between sleep and depression and to further explore the impact of sleep on depression in elite youth athletes. Second, we only used subjective measures to assess sleep and depressive symptoms, and we did not collect any qualitative data. However, these subjective measurements may be superior tools for screening the risk of sleep problems and depressive symptoms in large-scale athletic populations. Moreover, the PSQI and MEQ questionnaires have not been specifically validated for Korean adolescents, although these tools are widely used to assess sleep and chronotype in this group [39,40]. Third, because the study included only elite athletes from the Korea Next Generation Project, the sample size was relatively small for conducting multivariate analyses, such as subgroup analysis by sex. Further studies are needed to include larger and more diverse samples, incorporating participants from other countries, to generalize our findings. Fourth, several other factors that are known to affect sleep and mental health, such as training intensity and dietary habits [5,41], were not included in this study.

## 5. Conclusions

Our findings showed that among Korean elite youth athletes, poor sleepers, identified by high global PSQI scores, exhibited increased odds of having depressive symptoms. The PSQI components, including poor subjective sleep quality, prolonged sleep latency, sleep disturbances, and daytime dysfunction, demonstrated significant associations with depressive symptoms. These findings highlight the importance of managing sleep and mental health to minimize inhibiting factors and maintain optimal conditions for athletes. Therefore, coaches, sports physicians, psychologists, athletes, and their parents should actively apply evidence-based methods and strategies, such as napping behaviors and the use of recovery garments [42,43], to enhance sleep quality and mental well-being in these young athletes.

## Figures and Tables

**Table 1:** Characteristics of study participants according to sleep quality.

	All (*n* = 248)	Good Sleepers (*n* = 120)	Poor Sleepers (*n* = 128)	*p*-Value
Age, years	16.4 ± 0.1	16.0 ± 0.2	16.8 ± 0.1	<0.001
Sex, *n* (%)				
Male	129 (52.0)	65 (54.2)	64 (50.0)	0.512
BMI, kg/m^2^	22.3 ± 0.2	22.0 ± 0.3	22.6 ± 0.3	0.178
CES-D score	12.5 ± 0.4	10.4 ± 0.4	14.5 ± 0.6	<0.001
CES-D category, *n* (%)				
Depression symptoms (CES-D ≥ 16)	59 (23.8)	14 (11.7)	45 (35.2)	<0.001
Chronotype category, *n* (%) ^#^				0.589
Morning type (59 ≤ MEQ score ≤ 86)	1 (0.4)	1 (0.8)	0 (0)	
Intermediate type (42 ≤ MEQ score ≤ 58)	239 (98.0)	116 (97.5)	123 (98.4)	
Evening type (16 ≤ MEQ score ≤ 41)	4 (1.6)	2 (1.7)	2 (1.6)	
Length of sports career, months	80.8 ± 2.2	73.8 ± 3.2	87.4 ± 2.9	<0.005
Sports category, *n* (%)				<0.001
Handball	27 (10.9)	24 (20.0)	3 (2.3)	
Badminton	58 (23.4)	33 (27.5)	25 (19.5)	
Short-track skating	43 (17.3)	11 (9.2)	32 (25.0)	
Speed skating	29 (11.7)	9 (3.5)	20 (15.6)	
Figure skating	21 (8.5)	10 (8.3)	11 (8.6)	
Weightlifting	16 (6.5)	1 (0.8)	15 (11.7)	
Wrestling	24 (9.7)	15 (12.5)	9 (7.0)	
Cycling	30 (12.1)	17 (14.2)	13 (10.2)	

Values are the mean ± standard error or number (%). ^#^ Chronotype category: *n* = 244. CES-D, Center for Epidemiological Studies Depression Scale; MEQ, Morningness–Eveningness Questionnaire. χ^2^ tests were used for categorical variables and independent *t*-tests (parametric tests) or Mann–Whitney U tests (non-parametric tests) were used for continuous variables to assess the significance of differences between good sleepers and poor sleepers.

**Table 2:** Comparison of PSQI scores between groups with and without depressive symptoms.

	Without Depressive Symptoms (*n* = 189)	With Depressive Symptoms (*n* = 59)	*p*-Value
Global PSQI score	5.24 ± 0.19	7.22 ± 0.36	<0.001
PSQI components			
Subjective sleep quality	0.93 ± 0.04	1.34 ± 0.08	<0.001
Sleep latency	0.98 ± 0.06	1.47 ± 0.14	<0.005
Sleep duration	0.93 ± 0.07	1.10 ± 0.13	0.782
Habitual sleep efficiency	0.14 ± 0.03	0.14 ± 0.06	0.826
Sleep disturbance	0.98 ± 0.04	1.32 ± 0.09	<0.001
Sleep medication	0.05 ± 0.03	0.05 ± 0.03	0.645
Daytime dysfunction	1.23 ± 0.06	1.80 ± 0.10	<0.001

Values are expressed as the mean ± standard error. PSQI, Pittsburgh Sleep Quality Index. A one-way analysis of covariance was performed to assess the significance of differences between groups with and without depressive symptoms, after adjusting for age, sex, body mass index, sport type, and length of athletic career.

## Data Availability

The data that support the findings of this study are available from the corresponding author upon reasonable request.
